# Bisphosphonate-Induced Atypical Femoral Fractures in Pediatric Patients With Lipoprotein Receptor-Related Protein 5 (LRP5) Gene Mutation: A Case Report of Twin Patients

**DOI:** 10.7759/cureus.87984

**Published:** 2025-07-15

**Authors:** Mohamed Sarhan, Moustafa Aly, Paul Williams, Simon Humphry

**Affiliations:** 1 Trauma and Orthopedics, Swansea Bay University Health Board, Swansea, GBR

**Keywords:** atypical femur fracture, bisphosphonate therapy, mutation of lrp5 gene, paediatric orthopedics, pediatric genetics

## Abstract

Atypical femoral fractures (AFFs) are rare complications in children receiving bisphosphonate therapy for bone disorders. Early-onset osteoporosis in this population is often linked to monogenic bone diseases caused by genetic mutations. This study aims to highlight the risk of AFFs associated with bisphosphonate therapy in pediatrics and review the treatment approaches for these fractures. We report two cases of AFFs in nine-year-old identical twins with early-onset osteoporosis secondary to a lipoprotein receptor-related protein 5 (LRP5) gene mutation. Both had been receiving long-term zoledronic acid therapy. Each twin sustained a low-energy, left-side, transverse fracture of the proximal femoral shaft within a span of nine days. Radiological imaging revealed evidence of an impending fracture on the contralateral femur in both cases. The fractures were managed with intramedullary nailing using the Orthopediatrics Simple Locking IntraMedullary (SLIM^TM^, OrthoPediatrics Corp., Warsaw, IN) nail system, augmented with a lateral plate and screws. Sequential prophylactic fixation of the contralateral side was also performed using the same nail system. Radiographic evaluation at six weeks showed progressive callus formation, with complete union by 12 weeks. Clinically, both patients achieved full weight-bearing capacity without pain and returned to age-appropriate daily activities. No surgical complications, implant failures, or recurrent fractures were observed during follow-up. The described fixation techniques for the AFFs in pediatric patients proved to have a good functional outcome with full healing. Further studies are required to determine whether the risk of AFFs is primarily influenced by the underlying monogenic bone disease, the long-term use of bisphosphonates, the dosage regimens involved, or a combination of these factors. Contralateral limb imaging is recommended to identify potential impending fractures and ensure appropriate management.

## Introduction

Early-onset osteoporosis is often associated with gene mutations underlying monogenic bone disorders. Atypical femoral fractures (AFFs) have been reported in individuals with the following monogenic bone disorders and their associated genes: osteogenesis imperfecta (COL1A1/COL1A2), pycnodysostosis (CTSK), hypophosphatasia (ALPL), X-linked osteoporosis (PLS3), osteopetrosis, X-linked hypophosphatemia (PHEX), and osteoporosis-pseudoglioma syndrome (lipoprotein receptor-related protein 5 (LRP5)) [1]. Variants in the LRP5 gene are particularly associated with osteoporosis-pseudoglioma syndrome, characterized by severe osteoporosis and ocular abnormalities [2]. Mutations in the LRP5 gene can cause primary osteoporosis by reducing Wnt signaling activity, which is a critical pathway for bone formation and remodeling [3]. The LRP5 protein plays a role in the formation of retinal blood vessels and the regulation of bone mineral density [2,4].

Between the 1990s and 2000s, bisphosphonates (BPs) were approved for clinical use as leading therapies for several major bone-related diseases, including osteoporosis and skeletal-related events associated with bone metastases [5]. BPs are a class of calcium-binding drugs used to prevent bone resorption by selectively targeting and inhibiting bone-resorbing osteoclasts, thereby reducing bone turnover and preserving bone density [6]. However, there have been reports of atypical subtrochanteric and diaphyseal femoral fractures associated with long-term BP therapy [7]. AFF is a rare complication in children undergoing BP therapy for treating bone disorders [8].

We report AFFs in nine-year-old identical twins known to have LRP5 gene mutation-related osteoporosis treated with BPs. Each sustained an AFF on one side and had an impending atypical femur fracture on the contralateral side.

## Case presentation

Case 1

**Figure 1:**
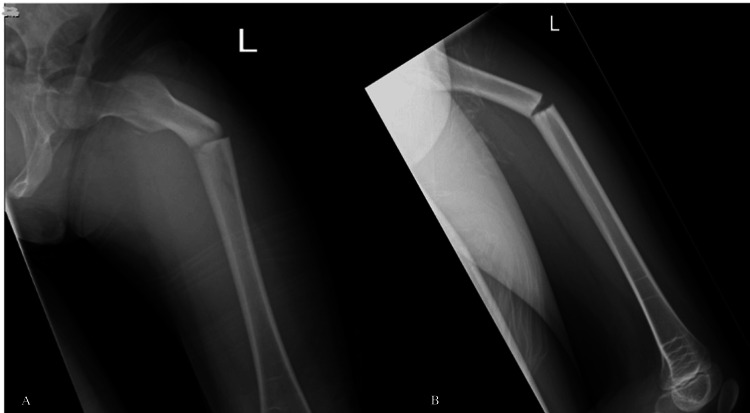
Anteroposterior (A) and lateral (B) radiographs of the left femur demonstrating an atypical diaphyseal femur fracture (Case 1)

A nine-year-old girl sustained an atypical femoral diaphysis fracture on the left side after falling from a trampoline in May 2024 (Figure 1). She had a history of ongoing prodromal thigh pain prior to the injury. The injury was a closed fracture with an obvious deformity of the left thigh. Neurovascular integrity was preserved, and there were no signs of compartment syndrome.

Case 2

**Figure 2:**
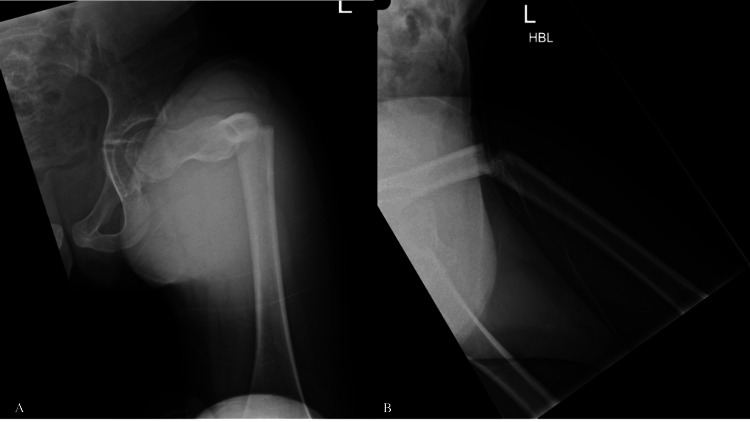
Anteroposterior (A) and lateral (B) views of the left femur showing an atypical diaphyseal femur fracture (Case 2)

The twin sister of case one, who also experienced ongoing prodromal left thigh pain before her injury, sustained an atypical left femoral diaphysis fracture (Figure 2) after performing a forward roll at school. This injury occurred nine days after her twin sister's fracture. The injury was closed, with an obvious deformity of the left thigh, and neurovascular function remained intact, without signs of compartment syndrome. She was admitted to our unit while her twin sister was still hospitalized.

In 2021, they were diagnosed with an LRP5 gene mutation following genetic testing conducted on them and their parents. Both had severe osteoporosis and visual abnormalities. They began receiving zoledronic acid infusions administered biannually by the metabolic bone team in Cardiff, starting in December 2021.

Treatment

**Figure 3:**
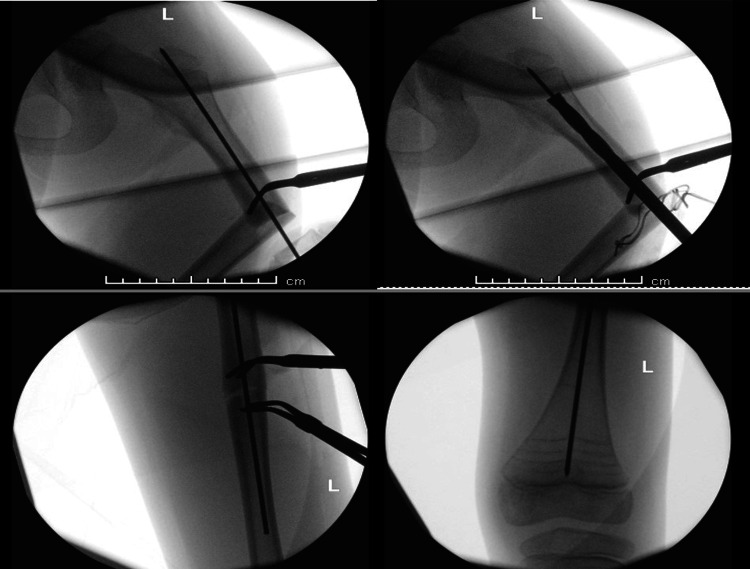
Intraoperative images for open reduction and retrograde guide wire introduction in left femur fracture (Case 1)

**Figure 4:**
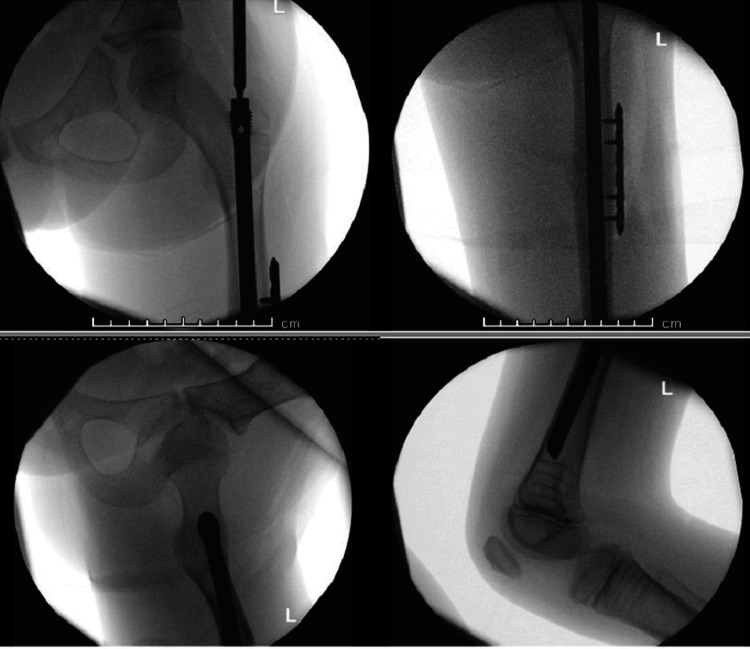
Intraoperative images after left femur fracture fixation with SLIM nail and plate, and screws (Case 1)

Each patient underwent intramedullary nailing using the Orthopediatrics Simple Locking IntraMedullary (SLIM^TM^, OrthoPediatrics Corp., Warsaw, IN) nail system [9], augmented with a lateral plate and screws for fixation of the fractured femur. The procedures were performed under general anesthesia and regional block, with prophylactic antibiotics administered at induction. With the patient in the supine position and continuous image guidance, a lateral approach to the femur was utilized, and the fracture ends were refreshed. A 2 mm guide wire for the SLIM^TM^ nail was introduced retrograde through the fracture site, and then the proximal lateral skin incision was made. While maintaining proper reduction and rotational alignment, the guide wire was advanced distally under fluoroscopic guidance (Figure 3). After canal reaming, a six-hole 2.7 mm lateral plate was applied and secured with four unicortical locking screws. A 6.4 mm SLIM^TM^ nail was then inserted while ensuring reduction is maintained, and the construct was finalized with the application of an end cap (Figure 4).

**Figure 5:**
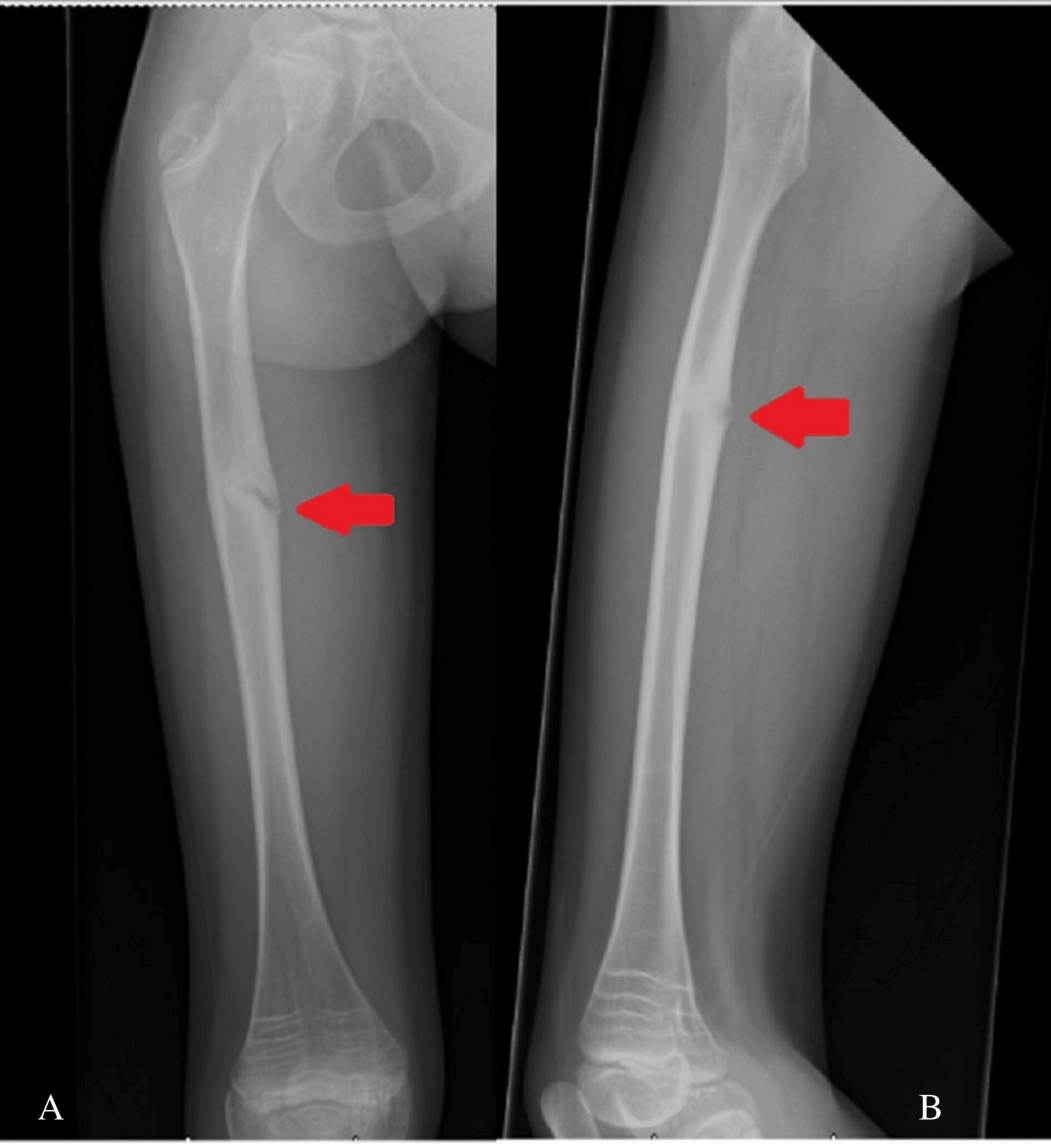
Anteroposterior (A) and lateral (B) views of the right femur showing an impending femoral fracture (indicated by red arrows, Case 1)

**Figure 6:**
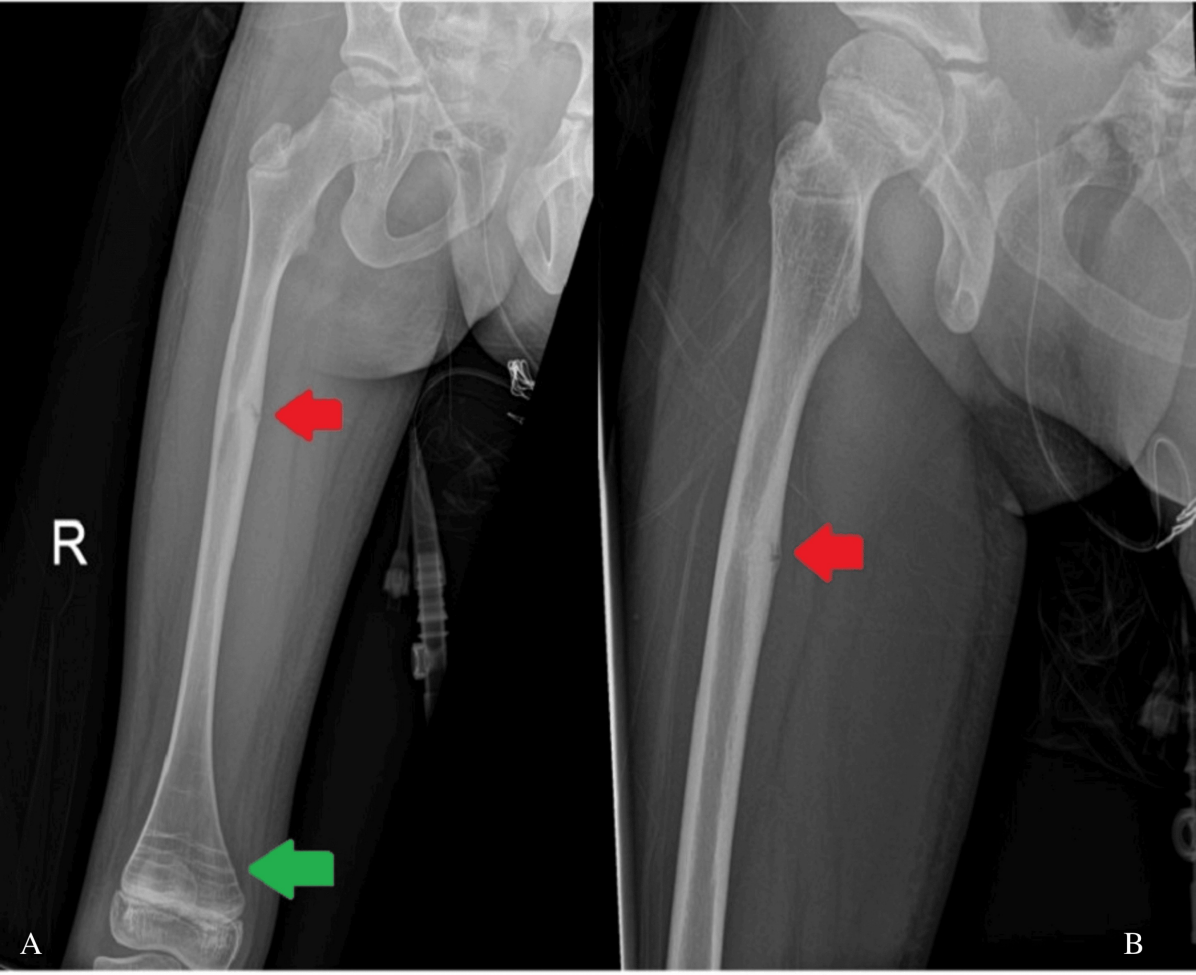
Anteroposterior (A) and lateral (B) views of the right femur showing an impending femur fracture (indicated by red arrows, Case 2) The femur distal metaphysis shows horizontal sclerotic lines, known as Harris lines (green arrow).

**Figure 7:**
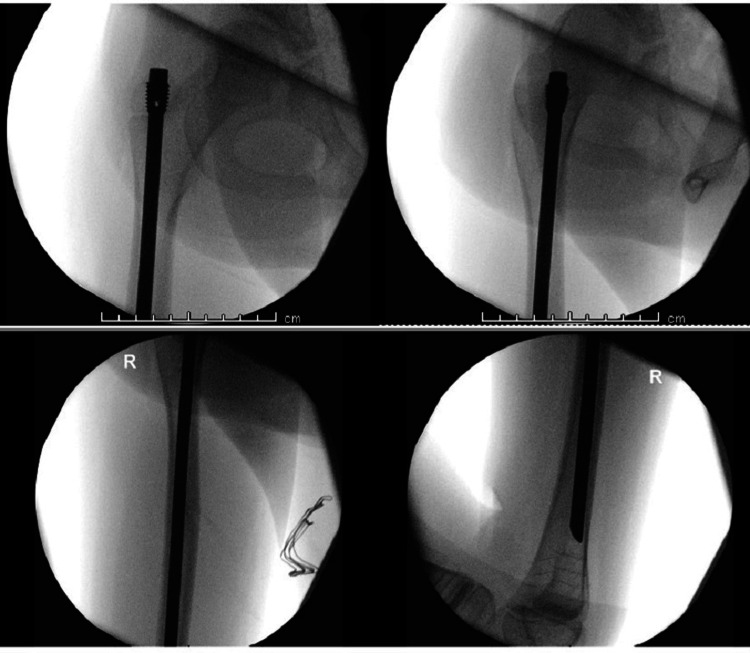
Intraoperative images after right femur prophylactic fixation with SLIM nail (Case 2)

During their admission, a right femur X-ray was performed to rule out a contralateral femur fracture and revealed an impending femoral shaft fracture in both patients (Figures 5, 6). Based on these findings, a prophylactic intramedullary fixation of the right femur was performed one week after fixation of the fractured side. The surgery was carried out using the same surgical setup as the initial procedure. A standard lateral proximal incision was made on the right thigh. A 2 mm guide wire was inserted while maintaining the position using a closed technique. Following canal reaming, a 6.4 mm SLIM^TM^ nail was inserted (Figure 7). The proximal threads engaged the greater trochanter, and an end cap was applied to secure the construct.

Outcome and follow-up

**Figure 8:**
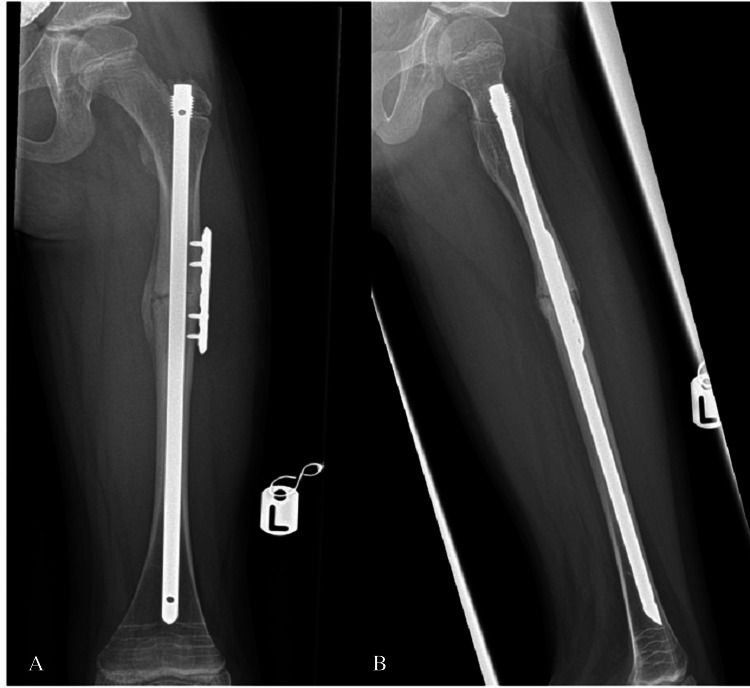
Anteroposterior (A) and lateral (B) views showing healing signs in the left femur fracture at six weeks postoperatively (Case 1)

**Figure 9:**
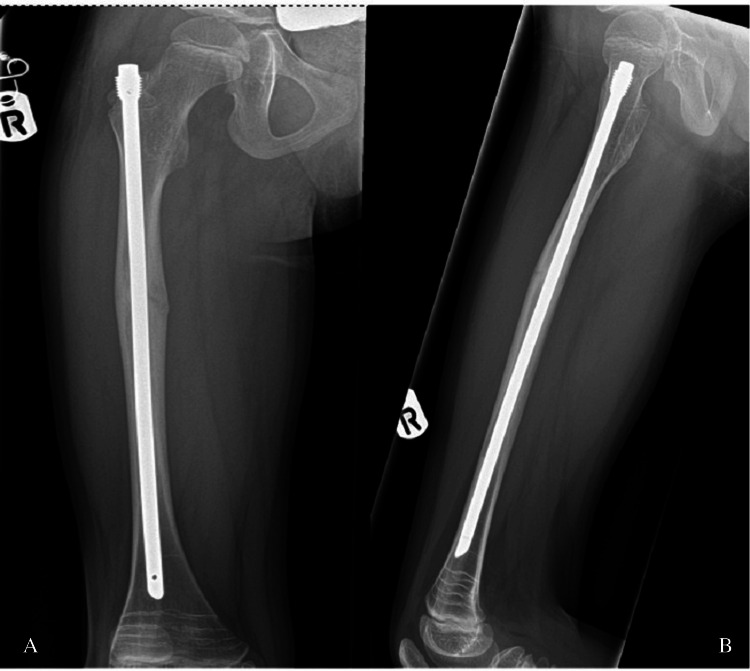
Anteroposterior (A) and lateral (B) views showing healing signs in the right femur fracture at six weeks postoperatively (Case 1)

**Figure 10:**
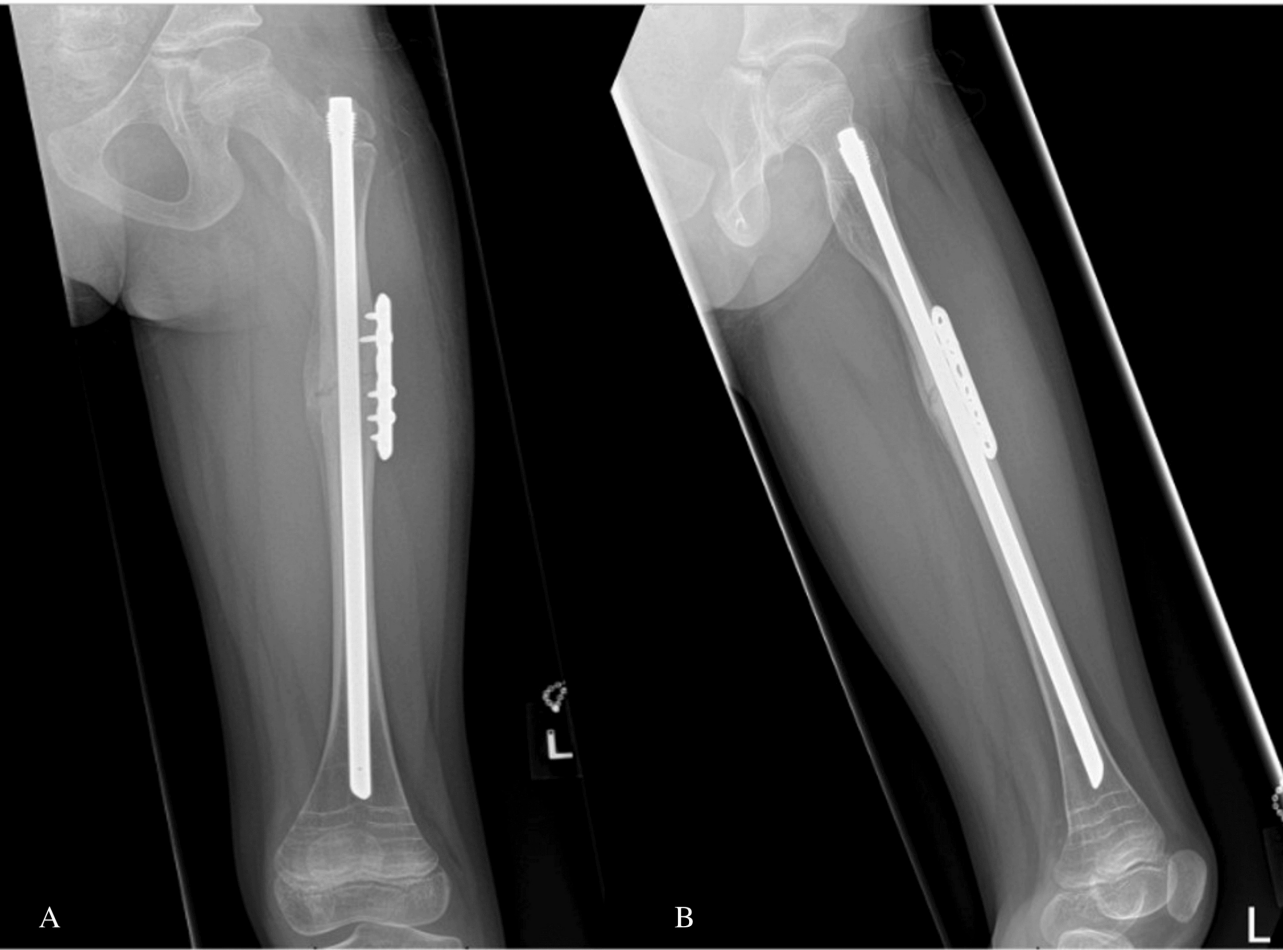
Anteroposterior (A) and lateral (B) views showing healing signs in the left femur fracture at six weeks postoperatively (Case 2)

**Figure 11:**
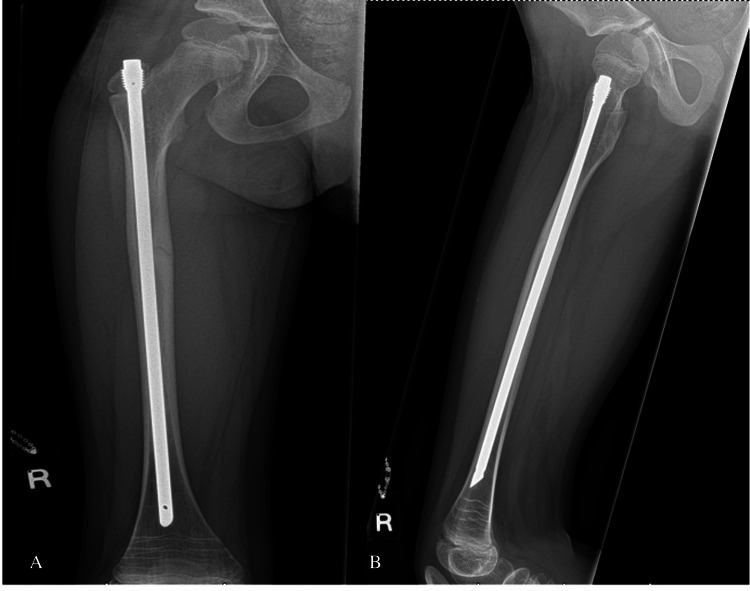
Anteroposterior (A) and lateral (B) views showing healing signs in the right femur fracture at six weeks postoperatively (Case 2)

Postoperatively, both patients were allowed full weight-bearing with crutches following fixation of bilateral fractures. The surgical wounds healed well with no complications observed. At the six-week outpatient review, both patients demonstrated a full, pain-free range of motion in their hips and knees, with no tenderness at the fracture sites. Neurovascular examination revealed no distal deficits. Radiological evaluation showed satisfactory signs of bone healing in both patients bilaterally (Figures 8-11).

## Discussion

AFFs have been categorized as stress or “insufficiency” fractures and linked to long-term anti-resorptive therapy, particularly BPs [10]. The American Society of Bone and Mineral Research (ASBMR) published a report on AFF case definition, which illustrated that an AFF should fulfill at least four of five major features: minimal or no trauma; originates at the lateral cortex and has transverse orientation; non-comminuted or minimally comminuted; complete fractures extend through both cortices and incomplete fractures involve only the lateral cortex; and localized cortical thickening at the fracture site [11].

The mechanism has been suggested to be the suppressive effect of BPs on bone remodeling, which leads to non-healing micro-cracks and ultimately to full-scale fractures. The localized cortical thickening, also typical for stress fractures, supports this hypothesis [12]. It remains unexplained why AFFs occur in only a minority of patients treated with BPs. In addition, it is still unexplained why these fractures also occur in patients who never used BPs [13]. While the pathogenesis of AFFs remains unclear, the detection of these fractures in individuals who have not been exposed to BPs and in those with inherited bone disorders has raised the idea that genetic factors may contribute to the susceptibility of AFFs [14].

There are some suggestions, like taking a break from BP therapy, which is referred to as a “drug holiday,” would decrease the risk of AFFs with no large increased risk of osteoporotic fractures; however, not all the authors agree on this [15].

Del Real et al. [14] suggested that the accumulation of some gene variants may contribute to determining the risk of AFFs in patients on anti-resorptive drugs. The individual susceptibility to AFFs seems to be affected by polygenic factors, as well as drug therapy. Further studies on these genes could help in determining which patients are at higher risk of developing AFFs when treated with anti-resorptive drugs. Given the polygenic origin suggested by the present and other studies, the use of polygenic risk scores can be considered [14].

Garcia-Giralt et al. [16] concluded that 33.3% of the patients of AFF fractures had genes belonging to the Wnt pathway; one of them was LRP5. However, it is difficult to discern whether these genes play a role in the pathophysiology of AFF in addition to their known role in low bone mass [16]. Moreover, Zhou et al. [13] found that LRP5 was mutated in two AFF patients with a diagnosis of monogenic osteoporosis, suggesting this putative dual role [13].

In view of these recent studies [13,14,16], the presence of genetic variants, such as the LRP5 mutation with monogenic bone disorder, must be considered as a contributing factor to AFFs, as well as BPs.

This paper describes two nine-year-old identical twins; each sustained an atypical femur fracture on one side and an impending atypical femur fracture on the contralateral side after being on BPs for treatment of LRP5 gene mutation-related osteoporosis. To our knowledge, this is the first study to report simultaneous bilateral atypical femur fractures in identical twins in this age group.

Given that these two cases involve LRP5 gene mutations associated with monogenic osteoporosis and a three-year history of BP therapy, the feasibility of utilizing genetic risk scores prior to initiating BP therapy should be investigated. Considering the risk of AFFs linked to genetic predispositions, implementing a regular X-ray screening protocol could be a valuable screening measure.

The development of a reliable scoring system for X-ray screening may be beneficial, incorporating factors such as family history, clinical symptoms, duration of BP therapy, and the underlying genetic condition. However, it is important to balance the need for regular imaging with the potential risks of excessive radiological exposure.

The decision to consider a nail exchange in these patients should be based on factors such as fracture healing, potential growth plate complications, or implant failure observed during follow-up assessments.

## Conclusions

BPs are effective in treating bone disorders in children; however, further studies are required to determine whether the risk of AFFs is primarily influenced by the underlying monogenic bone disease or the long-term use of BPs, the dosage regimens involved, or a combination of these factors. Contralateral and whole-limb long bone imaging is strongly recommended to identify potential impending fractures and ensure appropriate management. The above-described fixation techniques for the AFFs in pediatric patients proved to have a good functional outcome with full bone healing. Therefore, they can be considered in AFF management.

## References

[1] Nguyen HH, van de Laarschot DM, Verkerk AJ, Milat F, Zillikens MC, Ebeling PR (2018). Genetic risk factors for atypical femoral fractures (AFFs): a systematic review. JBMR Plus.

[2] Fabre S, Bourmaud M, Mabilleau G (2023). Lrp5 p.Val667Met variant compromises bone mineral density and matrix properties in osteoporosis. JBMR Plus.

[3] Korvala J, Jüppner H, Mäkitie O (2012). Mutations in LRP5 cause primary osteoporosis without features of OI by reducing Wnt signaling activity. BMC Med Genet.

[4] Huang W, Li Q, Amiry-Moghaddam M (2016). Critical endothelial regulation by LRP5 during retinal vascular development. PLoS One.

[5] Ebetino FH, Sun S, Cherian P (2022). Bisphosphonates: the role of chemistry in understanding their biological actions and structure-activity relationships, and new directions for their therapeutic use. Bone.

[6] Rogers MJ, Mönkkönen J, Munoz MA (2020). Molecular mechanisms of action of bisphosphonates and new insights into their effects outside the skeleton. Bone.

[7] Villegas RI, Melo-Durán S, Cevallos A (2024). Atypical femoral fractures due to the use of bisphosphonates. Experience of two institutions (Article in Spanish). Acta Ortopédica Mexicana.

[8] Albertson B, Polander T, Silva S (2021). Bisphosphonate associated atypical femur fracture and contralateral impending atypical femur fracture in a pediatric patient with osteogenesis imperfecta: a case report. WJO.

[9] (2025). SLIM™ - Simple Locking IntraMedullary System. https://www.orthopediatrics.com/products/slim-simple-locking-intramedullary-system-pega-orthopediatrics/.

[10] El Miedany Y (2022). Atypical femur fractures. New Horizons in Osteoporosis Management.

[11] Zhou W, van Rooij JG, Ebeling PR, Verkerk AJ, Zillikens MC (2021). The genetics of atypical femur fractures—a systematic review. Curr Osteoporos Rep.

[12] Vuorimies I, Mäyränpää MK, Valta H, Kröger H, Toiviainen-Salo S, Mäkitie O (2017). Bisphosphonate treatment and the characteristics of femoral fractures in children with osteogenesis imperfecta. J Clin Endocrinol Metab.

[13] Zhou W, van Rooij JG, van de Laarschot DM (2023). Prevalence of monogenic bone disorders in a Dutch cohort of atypical femur fracture patients. J Bone Miner Res.

[14] Del Real Á, Cruz R, Sañudo C (2024). High frequencies of genetic variants in patients with atypical femoral fractures. Int J Mol Sci.

[15] Black DM, Condra K, Adams AL, Eastell R (2022). Bisphosphonates and the risk of atypical femur fractures. Bone.

[16] Garcia-Giralt N, Ovejero D, Grinberg D, Nogues X, Castañeda S, Balcells S, Rabionet R (2024). Assessing the contribution of genes involved in monogenic bone disorders to the etiology of atypical femoral fractures. Hum Genomics.

